# Dynamics of double strand breaks and chromosomal translocations

**DOI:** 10.1186/1476-4598-13-249

**Published:** 2014-11-18

**Authors:** Olga V Iarovaia, Mikhail Rubtsov, Elena Ioudinkova, Tatiana Tsfasman, Sergey V Razin, Yegor S Vassetzky

**Affiliations:** Institute of Gene Biology, Russian Academy of Sciences, Moscow, 117334 Russia; UMR8126, Université Paris-Sud, CNRS, Institut de cancérologie Gustave Roussy, Villejuif, 94805 France; LIA1066 « Laboratoire franco-russe de recherche en oncologie », Villejuif, France; Faculty of Biology, M.V. Lomonosov Moscow State University, Moscow, 119992 Russia

## Abstract

Chromosomal translocations are a major cause of cancer. At the same time, the mechanisms that lead to specific chromosomal translocations that associate different gene regions remain largely unknown. Translocations are induced by double strand breaks (DSBs) in DNA. Here we review recent data on the mechanisms of generation, mobility and repair of DSBs and stress the importance of the nuclear organization in this process.

## Introduction

Many cancers are characterized by chromosomal translocations. First evidence of translocations and their possible role in cancer was put forward by German cytologist Theodor Boveri 100 years ago, in 1914 [1]. Translocations arise as a consequence of erroneous DNA double strand break (DSB) repair. The DSBs appear during physiological processes, such as recombination of immunoglobulin genes in the course of lymphoid cell maturation, in pathological processes and under the influence of external conditions such as oxidative stress and ionizing radiation (for review see [2]). A combination of several events is required for a translocation to take place: these include errors in DSB repair, spatial proximity of translocation partners and the capacity of damaged loci to relocalize within the nuclear space.

Currently, two hypotheses have been formulated to explain the proximity of damaged loci resulting in translocation events. The “contact first” hypothesis proposes that the translocation partner loci are proximal within the nucleus during the event leading to DSBs. The key prediction of this model is close spatial proximity of translocation partners [3]. This proximity may be permanent as in the case of RET and H4 loci [4] or temporary, as in the case of *c-myc* and immunoglobulin heavy chain (*IgH*) loci when *c-myc* dynamically colocalizes with *IgH* upon activation of B-lymphocytes [5]. This colocalization may occur within specific nuclear compartments, such as specialized repair foci (in yeast), transcription factories, replication foci, and others (for review see [6]).

Positions of the chromosomes in the nuclear space also affect the localization of potential translocation partners. Gene-rich and small chromosomes tend to be clustered in the center of the nucleus [7]; thus a probability of their direct contact is much higher than in the case of larger chromosomes. The “contact first” hypothesis postulates that the broken chromosome ends are immobile or have a reduced mobility thus maximizing the probability of the translocation of neighboring loci.

An alternative “breakage first” hypothesis postulates that chromosome ends with DSBs can freely move within the nuclear space and that collision of damaged loci on different chromosomes may lead to translocations. In this case, the probability of translocation may increase with the scale of DSB movement. This movement can be either directed or stochastic, resembling the Brownian motion of particles. The amplitude of stochastic movements might increase after the DSBs thus increasing the volume of the nuclear space scanned by the damaged locus. The probability of collision of distant loci followed by a translocation might also increase in this case. A combination of both directed and stochastic movements is also possible: the stochastic movement might bring the locus in contacts with a structure, e.g. a nuclear membrane, interchromosomal space or a perinucleolar region [8], and in this case the movement might become directed and follow encountered structure.

DSBs arise in cells under the influence of external factors, such as oxidative stress and ionizing radiation, but they are also associated with physiological processes such as DNA replication [9]. RAG1/2 and AID-induced DSBs in immunoglobulin genes are required for V(D)J recombination, somatic hypermutation and class switch recombination essential for creation of antigen repertory [10]. DSBs may also arise during viral infection [11]. In yeast and higher eukaryotes, multiple DSBs arise at the beginning of meiotic recombination [12].

In normal conditions, DSBs are repaired either by homologous recombination (HR) which requires the presence of the sister chromatid or by non-homologous end joining (NHEJ), a direct ligation of damaged DNA ends [13]. HR is a high fidelity mechanism, but it requires the presence of a sister chromatid or homologous sequences, therefore it is mostly used at late S/G2 phases of cell cycle and in meiosis. NHEJ is used in other cases, and it is error-prone. It may lead to both small errors at the DSB site and to translocation of large DNA regions (for review see [14]). In the following section we shall concentrate on DSBs and their repair that potentially lead to translocations.

The movement of DSBs in the nuclear space could be dependent on the break stability: both ends of a DSB may stay associated before the translocation. In case of the encounter of two neighboring loci with DSBs, a reciprocal translocation may occur. Alternately, a DSB may disturb the integrity of the chromosome. The ends of the DSB separate and the translocations events for each end become independent. In this case, the translocations will not be reciprocal. Repair kinetics and the time of persistence of DSBs also play a role in the mechanisms of translocation. Indeed, persistent DSBs are more likely to produce translocations (reviewed in [15, 16]).

### Current methods of detection of DSBs and their mobility

Currently it is not yet clear whether DSBs lead to an increased mobility of the damaged locus. The data both confirming this hypothesis [4, 17–21] and contradicting it [22–24] exist (reviewed in [25, 26]). This controversy may be due to several factors, i.e. significant differences between the model systems used and the epigenetic patterns of the models, various eukaryotic cell lines and yeast. The methods of introduction of DSBs, the number of induced DSBs, and the way to visualize the break and measure its mobility may also affect the conclusions. Other important factors to consider are the phase of the cell cycle, the DSB localization in the genome, chromatin context, the nature of the DSB, the presence of DNA adducts and the mobility of the locus in the absence of any damage and the mechanism of DSB repair. The role of some of these factors is reviewed in [27].

Translocations may be initiated by induction of a limited number of DSBs by ultrasoft X-rays, laser micro-irradiation, γ-irradiation, α-particles. DSBs may also be randomly induced by chemical agents, including topoisomerase poisons used in cancer therapy. Non-random DSBs can now be induced using meganucleases and TALEN nucleases. A direct comparison between results obtained using different types of DSBs is possible only in the case when the amount of generated DSBs and local DSB densities are comparable [28]. Even in this case the conclusions may be compromised by different nature of DSBs, i.e. the absence or the presence of protruding ends as well as the presence of covalently bound proteins in case of DNA topoisomerase II-induced breaks may play an important role in DNA mobility (see below).

DSBs can be easily detected in fixed cell preparations using antibodies against DSB-specific proteins, DNA repair proteins or by BrdU incorporation at the DSB site. Of course, it is currently impossible to follow the movements of a single damaged locus in a particular fixed cell. The dynamics of DSBs and/or repair loci can only be studied in this case by comparing the position of the introduced break(s) to some control points at different time points followed by statistical analysis. The mobility of the damaged loci may also be followed by induction of regularly spaced DSBs. This can be done by using an irradiation-masking microgrid that allows to obtain a regular pattern of DSBs and evaluate the mobility of DSBs vs. this regular pattern [20]. The dynamics of a DSB movement may also be studied by comparing its position to positions of some specific loci using in situ fluorescent hybridization (FISH).

A progress in time lapse microscopy allows now to study the dynamics of DSBs in living cells in real time [27, 29]. Several approaches are used to visualize DSBs in living cells: the cells can be transfected by vectors expressing one or several DNA repair factors fused to fluorescent proteins. These factors assemble at the DSB site and thus label the DSB. The dynamics of the DSB labeled this way can be followed during several hours. Chromatin fluorescently labeled with GFP-H2B histone may serve as a reference point to detect DSB movement [17]. DSBs can be oriented along a straight line; in this case the disturbance of the initial pattern suggests that DSBs move within the nuclear space. The use of photo-activated fluorescent proteins allows to introduce the DSBs and to visualize them simultaneously [17].

The most sophisticated approach allows labeling *in vivo* a unique site in the genome with a fluorescent probe, to introduce a DSB and then follow the DSB movement. This can be done by integrating into the genome a construct containing the I-SceI recognition site surrounded by *lacO* and/or *tetO* sites [22, 30]. In cells producing I-SceI and the corresponding fluorescently tagged repressors, the integrated locus can be specifically cleaved while being fluorescently labeled. This allows to visualize the trajectory of the DSB up to the moment when it is repaired. This approach might be also used to study translocations when two potential translocation partner loci are tagged with different fluorochromes [30]. Unfortunately, the DSBs induced by I-SceI are just a simplified model of what is happening in real life; moreover the presence of multiple *lacO* and/or *tetO* sites induces formation of heterochromatin structures and affects gene expression [31, 32]. The results obtained in such model systems may not directly reflect real-life situation of genome damage and DNA repair.

DSB mobility is estimated using mean square displacement (MSD); MSD = Δx^2^(t), where x reflects the path of the locus after DSB induction and t is time after DSB induction. MSD also depends on the viscosity of the medium via a diffusion coefficient. MSD analysis allows to determine the volume of nucleus scanned by the locus with the DSB. If the mobility of the locus is restricted, the MSD value rapidly reaches a plateau.

Relative mobility of two loci can be characterized by mean square change in distance (MSCD). This method implies that the mobility of both loci has a similar pattern, which is not always the case [24, 26].

### DSB mobility in yeast: a role in DNA repair?

There is a significant difference between the behavior of DSBs in yeast and in higher eukaryotes: in yeast, DNA is repaired in specific nuclear compartments, repair foci. The damaged loci may be relocated into these repair foci as observed in [33]. Higher eukaryotes do not possess a specific repair compartment, therefore the damaged locus remains relatively immobile and repair factors move towards it to assemble on the DSB site. The increase in mobility of the damaged locus in this case occurs mostly because of the increase of the amplitude of Brownian motion and local chromatin decompaction at DSB site. Large-scale and directed movements are rare events that occur in particular areas and in case of complex DNA damage [6].

A relative consensus exists on the mobility of damaged loci in yeast: several DSBs assemble at Rad52-containing repair foci [33], suggesting that the damaged loci are highly mobile in the nuclear space and form clusters. Indeed, introduction of DSBs is accompanied by the increase in mobility of damaged loci [22, 24]. A four-fold increase in locus mobility was observed upon introduction of a DSB, but not a single-stranded break in haploid cells, and a two-fold increase, in diploid cells [24]. The damaged locus scanned ~30% of the nuclear volume in diploid cells [22, 24], and 47%, in haploid cells [22]. The undamaged locus could scan ~12% of the nuclear volume; this did not depend on the position of the target locus in the genome. In some cases, a DSB in one allele led to an increased mobility of the undamaged allele [24], in other cases, no effect on the mobility of undamaged loci was observed [22]. Increased mobility of the damaged locus might be specific for diploid cell and required for the sister chromatid search and repair.

Large-scale movement of damaged loci is specific for persistent DSBs. These DSBs are particularly dangerous for the cells. In yeast, persistent DSBs move towards the nuclear periphery through association with Mps3, telomerase and proteins of the nuclear pore complex [34]. DSBs in the telomeric regions are also sequestered in the nuclear periphery [35, 36]. It is yet unclear why persistent DSBs are recruited to the perinuclear region, possibly this recruitment is required to isolate them or delay their replication. This relocalization may also represent an attempt to repair these DSBs in a specialized compartment using alternative repair mechanisms. Mobility of DSBs in yeast cells is mediated by several protein factors: key repair enzymes RAD51, Sae2, RAD54, Mec1, and RAD9 [22, 24, 37]. Recruitment of a persistent DSB to the nuclear periphery also depends on H2A.Z [38]. It is unclear whether DSB relocation is an ATP-dependent process.

Importantly, the damaged locus moves as a whole and the damaged ends remain associated within the locus [33, 39, 40]. DSB stabilisation in this case is mediated by the MRX complex and exonuclease I that processes DSB ends before DNA repair [41].

### DSB mobility in higher eukaryotes

In contrast to yeast, the published data on mobility of DSBs in higher eukaryotes are quite controversial. Mobility of DSBs induced by X-rays or γ-irradiation did not differ from that of intact loci [18–21], reviewed in [25]). In most cases, very restricted movement of damaged loci was observed (with a diameter less than 0.5 μm, compare with an average diameter of the nucleus of 10 μm). Large scale-movements (>5 μm) were observed in a minority of nuclei (<2%) with altered nuclear morphology. The mobility of damaged loci did not depend on the used irradiation source and the number of induced DSBs [19]. Similar results were obtained in cells irradiated with an argon laser (364 nm) where DSBs were immobile and did not form clusters [17]. A local ATP-dependent decondensation of chromatin was observed at DSB sites in this case.

The majority of the I-SceI-induced single DSBs also did not change their position in the nuclear space [21]. It should be noted that restricted mobility of the locus in this case might be due to the presence of lacO binding proteins at the DSB site. The large size of the resulting complex might decrease the diffusion of the complex in the nucleus. Stability of the I-SceI-induced DSBs was independent of NBS1, MRN complex, SMC1 and H2AX. At the same time, the absence of Ku80 led to an increase in a number of cells where the DSB ends were dislocated, moreover, Ku80 knockdown increased the mobility of the damaged locus from 50 to 80 nm/min [21].

The opposite results on relocalization and clusterization of loci with DSBs were obtained by Aten et al., [23]. Cells were irradiated with α-particles so that DSBs were localized along a straight line, so that any subsequent DSB movement would disturb the initial linear pattern. The DSBs were detected by immunostaining with antibodies against phosphorylated H2AX. Alteration of the linear pattern could already be observed five minutes after DSB induction. DSB clustering was observed, mostly in G1 cells, while in S and G2 phase cells no clustering was revealed, but a part of damaged loci moved at distances up to 2 μm. Cells, deficient in one of repair pathways (HR or NHEJ) had a similar pattern of DSB mobility. Inactivation of XRCС-3 and inhibition of DNA-PK did not affect clustering of damaged loci [42], suggesting that relocalization and clustering of DSBs are not linked to DNA repair. Indeed, repair factors such as MRN complex, Ku80 and DNA-PK mostly ensure the DSB stability [43], with the exception of Rad51 which is capable to actively relocate chromatin fibres [44]. Below we shall consider two other important players in DSB mobility, large-scale nuclear organization and chromatin.

### DSB mobility and chromatin

Chromatin context in the damaged locus may significantly affect DSB mobility. Histone variant incorporation, histone post-translational modifications, and ATP-dependent chromatin remodelling - three major strategies for chromatin manipulation - may occur at DSBs (reviewed in [45, 46]). Histone variants (H2AX and H2A.Z), histone post-translational modifications (acetylation, phosphorylation, methylation and ubiquitination) and chromatin-remodeling complexes (INO80, SWR1, SWI/SNF, RSC and NuRD) are important and direct players in cellular responce to DSB. Changes of the chromatin status of the damaged locus may lead to two consequences: signaling and decompaction/remodeling of the chromatin fiber to increase the accessibility of the DSB for the repair machinery. Indeed, nucleosome eviction and sliding as well as exchange of histones in chromatin are required for DSB repair [45, 46].

**Figure 1:**
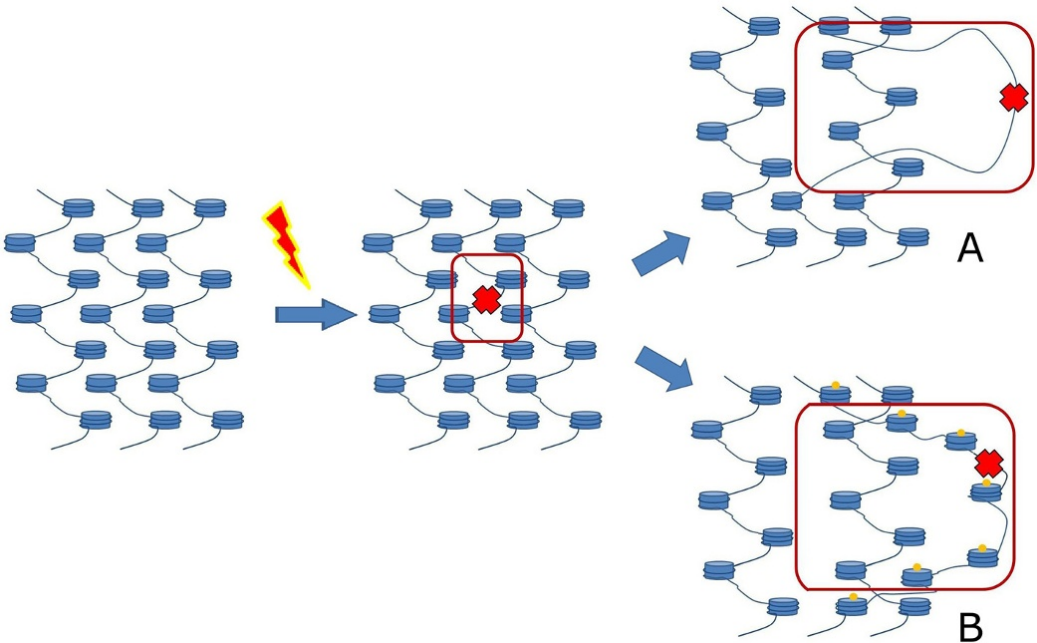
**Introduction of a DSB may increase chromatin mobility at DSB by formation of a nucleosome-free loop (A) or by chromatin decompaction (B).** Acetylated histones are marked with yellow dots.

How chromatin remodelling at DSB can affect the locus mobility? This subject has been extensively studied during the past few years (for review see [46–49]). Chromatin remodeling is necessary both for NHEJ and HR. In case of HR, active nucleosome eviction occurs at DSB followed by formation of single-stranded DNA. Nucleosome-free DNA has a higher mobility than chromatin (Figure 1). In yeast, deletion of arp8, which impairs INO80-dependent remodeling, leads to decreased mobility of a DSB [50]. In higher eukaryotes, a p400 SWI/SNF ATPase of the TIP60 complex provokes nucleosome destabilization around DSB [51]. At the same time, inhibition of the major chromatin remodelling factor at DSBs, Tip60 did not affect DSB mobility [52].

Another factor that can potentially affect DSB mobility is chromatin decompaction in the damaged locus (Figure 1). Chromatin decompaction after a DSB is accompanied by the changes in chromatin fibre mobility, increase in the volume occupied by the locus and thus an increase in passive mobility of the damaged locus. On the other side, chromatin decompaction may not be accompanied by increased DSB mobility [17].

Phosphorylation of the H2AX histone is one of the early responses of the cell to DNA damage. Phosphorylated H2AX (γH2AX) is spread over several megabases on both sides of a DSB, and attracts the MRN complex. This is followed by accumulation of 53BP1, RAD51, Nbs1, BRCA1 and chromatin remodeling complexes (INO80 и SWR1) at the DSB site (reviewed in [53, 54]). Besides this, H2AX phosphorylation changes the folding of a chromatin fiber by destabilizing the interaction between DNA and the H2AX-H2B dimer [55]. This results in formation of a relaxed chromatin domain where the damaged locus can have an increased mobility. Another histone variant, H2A.Z participates in relocalization of non-repaired DSBs to the nuclear periphery, where the DSBs are sequestered. The DSB relocalization is dependent on sumoylation of the C-terminus of H2A.Z [56].

Unfolding of a damaged locus is also due to DSB-induced post-translational modifications of all core histones (reviewed in [45]). Histone acetylation plays an important role in formation of a relaxed chromatin domain and an increase in DSB mobility. In yeast, DSBs lead to rapid acetylation of H4K16 followed by decompaction of the 30 nm chromatin fiber or other higher-order chromatin structures. This is followed by acetylation of other H4 residues (H4K5, H4K8ac, H4K12) that further relax chromatin. Epigenetic modifications at the original sites of DSBs showed local chromatin decondensation manifested by increased H4K5 acetylation and decreased H3K9 dimethylation [18, 57]. Decondensation of chromatin near DSB is accompanied by accumulation of acetylated H4K5 and H4K12, these signal partially coincided with the region enriched in γH2AX. Moreover, heterochromatin-associated persistent DSBs have a lower mobility than rapidly repaired DSBs [58]. An alternative model predicts higher mobility of heterochromatin-associated DSBs since establishment of a repair-competent focus may require its relocation into a “chromatin hole” where chromatin is relaxed [6]. The data on the role of chromatin in DSB mobility are quite controversial. Global changes in chromatin organization after cell treatment with inhibitors of DNA methylation, histone acetyltransferases and histone deacetylases significantly decreased the mobility of damaged loci and their clusterization [58]. At the same time, inhibition of the major chromatin remodelling factor at DSBs, Tip60 did not affect DSB mobility [52].

It is likely that a specific chromatin organization at and around the DSB site regulates repair by creating a specific compartment that coordinates DNA repair. The increase in DSB mobility may be due to gradual restructuring of chromatin in this repair-competent compartment. The probability of translocation may depend not on the collision of broken chromosome ends, but rather on a collision and fusion of repair-competent chromatin domains. The latter seems more likely. If these chromatin domains are structured, their fusion in a single compartment makes translocation a highly probable event.

### DSB mobility and large scale chromatin organization

Multiple DSBs are particularly dangerous since they may lead to translocations, but even isolated DSBs may produce massive damage to cell if they are located in regions that ensure structural and functional integrity of the genome. When a limited number of DSBs are introduced in DNA, the mobility of the damaged locus may be limited by a chromatin domain or a subcompartment, and thus depend on chromatin organization and the genome architecture. Currently there are several models of nuclear organization, most of these models predict the existence of chromatin domains separated by specific border elements (for review see [59, 60]). A nuclear matrix model predicts the existence of ubiquitous nuclear skeleton [60], some authors propose that the nucleus is structured by lamin-associated domains [61], and recent genome-wide chromatin conformation studies put forward the model of organization of nucleus into globular domains separated by linkers [62, 63]. Interestingly, all these models imply a key role of certain proteins in structuring the nucleus [64–67]. Lamin is one of these proteins and it also participates in ensuring the stability of DSBs [68]. It is likely that locus mobility is restricted to a specific topological domain, where folding of its elements inhibits the mobility of damaged loci. This aspect of DSB mobility remains largely unknown. Large-scale DSB movements may be mediated by molecular motors (nuclear actin and myosin), although currently there are no experimental data supporting this point of view.

**Figure 2:**
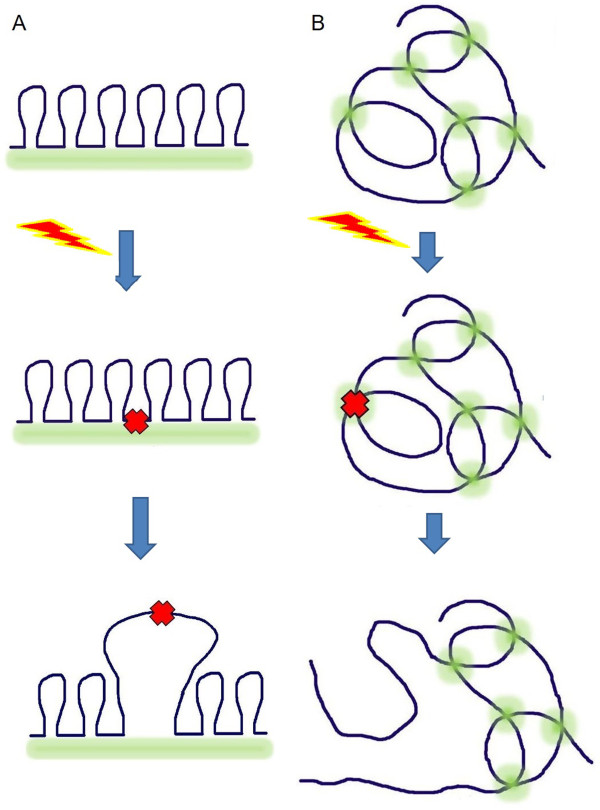
**Introduction of a DSB may perturb higher-order chromatin organization by targeting nuclear matrix attachment sites (A) or chromatin hub organizer sequences (B).**

When multiple and clustered DSBs are introduced into DNA, DSBs in key regions responsible for chromatin folding may destabilize the nuclear organization, thus greatly increasing the DSB mobility (Figure 2). These key regions may include the sites of DNA attachment to the nuclear matrix or regions involved in interaction in a chromatin globule (Figure 2). Such clustered DSBs are produced by high-energy α-particles and energy transfer induced by α-particles may indeed destabilizes nuclear structures and thus induce DSB clusterization. At the same time, data obtained on cell irradiated with low-energy γ-irradiation confirmed the observations obtained with α-particles [58]. DSBs were tagged in this case with 53BP1-GFP tagged protein. γ-irradiation induced formation of 53BP1-GFP foci and mobility of intact and damaged loci were studied using time lapse microscopy. A two-fold difference was observed in the nuclear volumes scanned by the damaged and the intact loci when DSBs were induced by γ-irradiation, and a three-fold difference was observed when DSBs were induced by topoisomerase II inhibitor etoposide. Inhibition of transcription did not affect the mobility of the damaged locus while ATP depletion decreased the mobility by 34%. The authors proposed that the damaged locus mobility was due to local chromatin decondensation and formation of a relaxed chromatin domain at DSB sites.

### Mobility of DSBs induced by topoisomerase II inhibitors

DNA topoisomerase II is an essential enzyme regulating DNA and chromatin topology. DNA topoisomerase II functions by forming a covalent complex with DNA, introducing a DSB and resealing the cleaved molecule [69, 70]. DNA topoisomerase II is an excellent target in cancer therapy [71, 72]. Topoisomerase poisons fix the covalent complex and inhibit religation of a DSB [69]. Stalled DNA-DNA topoisomerase II complex is then converted into a DSB and cell death followed DSB accumulation [72, 73]. Unfortunately, while being powerful and efficient anticancer drugs, topoisomerase poisons also induce secondary leukaemia due to high-frequency induction of translocations [74–76]. The frequency of these translocations obviously should depend on repair errors, on spatial proximity of the translocation partners and on mobility of the damaged loci.

Chromosomal breakpoints are clustered in so-called breakpoints cluster regions (*bcr*) in topoisomerase II poison-induced translocations [77, 78]. In normal conditions, DSBs including topoisomerase II-induced ones are repaired either by NHEJ or by HR [79–82]. Translocations arise from the NHEJ, and a specific type of NHEJ that is activated in case of DSB persistence might be responsible for the majority of translocations [83]. The translocations occur when correct joining of the DSB ends is impossible for some reason, e.g. high damage complexity, rapid destabilization of the DSB site and disjoining of the damaged ends or defects in DNA repair. Etoposide can trigger illegitimate recombination and translocations [82, 84].

Approximately 20-30% of therapy-related hematologic diseases contain balanced chromosomal translocations [85]. Treatment-related translocations mostly lead to expression of chimeric genes involved in regulation of haematopoiesis [86]. In many cases, these translocations concern *MLL* and *AML1* genes that translocate with a wide range of partners (http://atlasgeneticsoncology.org/) [78]. Currently there are no serious arguments in favour of the “contact first” or the “breakage first” hypotheses to explain the mechanism of these translocations. Analysis of nuclear localization of *AML1* and its most frequent translocation partner *ETO1* demonstrated that their colocalization frequency increased from 4.8% to 9.9% after treatment with topoisomerase poison etoposide [87, 88]. The frequency of colocalization of *MLL* and its partners in the nuclear space was shown to be inversely proportional to the *MLL/AF4* and *MLL/ENL* translocation frequency [89]. It has recently been shown that the frequency of *MLL* colocalization with its translocation partners *AF9* or *AF4* in one transcription factory did not exceed 2-3%, being almost a random event [90]. 3D FISH analysis of *MLL* locus revealed several interesting observations: at least in 17% of etoposide-treated cells, topoisomerase II-induced DSB was converted into a chromosomal break with two DSB ends being distant in the nuclear space; the frequency of this situation increased when etoposide was removed and the cell could repair the DSB [8]. Position of these breaks was significantly different from that of non-damaged loci: immediately after etoposide treatment, the DSB in MLL locus was localized within the chromosomal territory of chromosome 11, but after 1 hour chase, ~9% of the damaged loci moved outside their chromosomal territory, this behaviour was MLL-specific, other genes remained confined to their chromosomal territories after etoposide treatment [8].

Interestingly, a single *bcr*-targeted DSB in the *MLL* gene introduced by a Zn-finger nuclease was also converted into a chromosomal break [91]. Inhibition of DNA-PK increased the probability of dissociation of the damage locus ends four-fold. Unfortunately it is difficult to directly compare the mobility of a single nuclease-induced DSB and multiple DSBs induced by etoposide. Interestingly, the Zn-finger nuclease induced DSB in the *bcr* did not induce translocations [91], suggesting that a single DSB is not sufficient. Moreover, the probability of translocation may also increase in case of problems with DNA repair of complex DSBs. Topoisomerase poison-induced DSBs that arise in the *MLL bcr* may be clustered and heterogeneous, some are introduced by topoisomerase II, leaving DNA end bound via the topoisomerase II subunits; these can be converted into DSBs after ubiquitin-26S proteasome proteolysis of topoisomerase II [92, 93]. *The bcr* region is also a target of apoptotic nucleases, and apurinic and apyrimidinic sites may arise in the region from oxidative stress induced by etoposide [94]. Such complex clustered damage could be difficult to repair and leads to DSB persistence. Non-repaired DSB converted into a chromosomal break is a serious danger to the genome integrity.

Importantly, most translocations concerning *MLL* are reciprocal [95], and only a DSB that was not converted into a chromosomal break can produce a reciprocal translocation; thus large-scale mobility of the damaged locus may not give rise to translocations or produce non-reciprocal translocation that may lead to secondary leukaemias.

### DSB mobility and reciprocal vs. non-reciprocal translocations

An interesting insight on mobility of DSBs might be obtained from the analysis of primary translocations in cancer. Indeed, reciprocal translocations occur when both partner loci have DSBs that are not converted into a chromosomal break. Such translocations are characteristic for Non-Hodgkin lymphomas and leukaemias.

DSBs followed by chromosomal breakage mostly produce non-reciprocal translocations. Primary non-reciprocal translocations are characteristic of epithelial tumours [96] and solid tumours, e.g. renal cancer [97]. It must also be noted that the frequency of non-reciprocal translocations increases with progression in most types of cancer, but discussion of this topic is out of scope of the present review.

Therefore, a caution must be taken when applying results on DSB mobility obtained on one cell line to other cell types and tissues. Moreover, some results that we have reviewed above have been obtained on cancer cell lines that might be prone to specific types of translocation.

Other factors might induce translocations, e.g. telomeric dysfunction provokes appearance of non-reciprocal translocations in epithelial cells [98]. Telomere shortening might increase the DSB mobility and thus lead to chromosomal breaks at DSBs. The appearance of multiple translocations after a genotoxic stress may be linked to an increased DSB mobility and clusterization [99].

## Conclusions

Results obtained with different DSB models in higher eukaryotes are often inconsistent. Indeed, single DSBs are rarely converted into a chromosomal break. In this situation, an increase in the locus mobility is mostly linked to chromatin decompaction [46] that increases diffusion of the damaged loci. Multiple and clustered DSBs are frequently converted into a chromosomal break because repair mechanisms are not efficient in this case; alternately, multiple DSBs might disturb the nuclear architecture (nuclear matrix, lamin-associated domains or chromatin globules). This global disaster might delocalise both damaged and non-damaged loci and greatly increase the DSB mobility.

Studies of DSB mobility, its mechanisms and factors are important for better understanding of the mechanisms of translocations. A directed reduction of DSB mobility upon X-ray irradiation or DNA-damaging drug treatment may reduce a risk of secondary translocations in cancer.
